# Treatment of Trachoma by Expression

**Published:** 1892-02

**Authors:** William H. Baldinger

**Affiliations:** Galveston


For Daniel's Texas Medical Journal.

### TREATMENT OF GRANULAR LIDS OR TRACHOMA BY EXPRESSION.

WILLIAM H. BALDINGER, M. D., GALVESTON.

NEVER BEFORE, in the history of ophthalmology, has such active interest occurred in the treatment of granular lids as at the present time. I need offer no apology for touching on a topic so frequently discussed of late, for the simple reason, this disease has always been a source of much vexation to the practitioner in general and ophthalmologist in especial.

Heretofore many methods have been tried, and in turn put aside, and once more reliance placed alone in sulphate of copper.

With the revival of expression of granular masses, in suitable cases, by Dr. Gruening, of New York, the impetus for mechanical methods, an era of devising forceps for expression began. Trachomas previous to this time proving troublesome and requiring many months of treatment, were discharged after one ex-expression-sitting, followed by application of copper stick for a month or six weeks.

This was a decided advance. These effective operations were done with an ordinary flat-bladed forceps.

Following rapidly on this series of operations, such eminent specialists as Knapp, Noyes and a host of followers, devised forceps of various shapes and designs, each having some one or other advantage over the other, yet no more effective than that of Gruening in final result.

No matter how explicit as to the advisability of a method and when and how to operate, a rational method is defeated by abuse or wrong application of same.

After having witnessed numerous operations for two years in the various clinics and special hospitals in New York city (and several cases in private) I am fully convinced that in suitable cases, expression properly done, no matter by whose particular device of forceps it is accomplished, will shorten the treatment of granular lids manifold, and terminate the case successfully, to the satisfaction of both patient and practitioner.

